# Discordance in selected designee for return of genomic findings in the event of participant death and estate executor

**DOI:** 10.1002/mgg3.274

**Published:** 2017-01-16

**Authors:** Jessie L. Goodman, Laura M. Amendola, Martha Horike‐Pyne, Susan B. Trinidad, Stephanie M. Fullerton, Wylie Burke, Gail P. Jarvik

**Affiliations:** ^1^ Division of Medical GeneticsUniversity of WashingtonSeattleWashington; ^2^ Department of Bioethics and HumanitiesUniversity of WashingtonSeattleWashington; ^3^ Department of Genome SciencesUniversity of WashingtonSeattleWashington

**Keywords:** Executor, family communication, genomic sequencing, return of results

## Abstract

**Background:**

Legal and ethical questions arise regarding disseminating genetic research results to family members in the event of a research participant's death; failure to return or return to legal next of kin or estate executor may not reflect participant desires. We sought to determine participant preferences for whether and to whom they would like their data released in the case of their death prior to receiving genomic results, focusing on whether the person selected was also their estate executor.

**Methods:**

The University of Washington NEXT Medicine Study of the Clinical Sequencing Exploratory Research program previously reported participant preferences regarding designating an individual to receive genomic results in the event of death, including whether they want results shared, and if so, with what person. Participants were also asked whether this designee is executor of their will or estate.

**Results:**

To date, 61 individuals were asked about the concordance of their study designee and legal representative: 42 (69%) reported having a will or estate plan and of these, 14 (33%) chose someone other than their executor to receive their results. For the 14 who chose someone other than their estate executor to receive genetic results, 12 (86%) chose a family member, typically a biological relative, as their designee. Those with a different genomic designee than their executor were less likely to be partnered (*P* = 0.0024). For those partnered participants without an estate plan, spouses were not always chosen for return of genomic results.

**Conclusion:**

For one‐third of our participants, the individual deemed most appropriate by the participant to receive their genomic results was not the executor. In the absence of an explicit designation, HIPAA may prohibit access to genomic results to persons other than the executor; hence asking for designation at the time of study enrollment (or initiation of clinical testing) is important.

## Introduction

The integration of genetic and genomic data into clinical medicine has sparked important questions regarding how and what results are provided to patients and research participants (Jarvik et al. 2014). Exome and genome sequencing may generate both diagnostic and clinically actionable additional results (sometimes called incidental or secondary results) that may not have been predicted based on a personal or family history. Genetic medical information differs from most other family medical history information in potentially predicting significant risk for biological relatives and informing testing strategies. In general, a genetic test result in an affected family member is most informative; results for unaffected family members can be difficult to interpret in the absence of results from an affected family member. These characteristics make genetic information obtained in a relative of potential high interest to family members. Patients and research participants are encouraged to share their results with family members, but in some cases and of particular concern for those with cancer, they may die before results can be returned. This poses important questions about whether, and to whom, to return findings.

The Health Insurance Portability and Accountability Act's (HIPAA's) Privacy rule section 164.502 (g) (4), for HIPAA covered entities, allows only a person's personal representative, generally an estate executor, to receive protected health information upon a person's death (Amendola et al. 2015). If there is no designated representative, state law most often defaults to the next of kin, which may be a spouse. This person may not be aware of the existence of results of interest and not seek them. Further, this may not be the most appropriate person to communicate sensitive health‐related information to relatives. In the case of a spouse, they are not personally at risk from any clinically relevant genetic results.

While it is routine for clinicians to encourage patients to share their genetic information with relatives (Offit et al. 2004), research studies do not always have an established framework to encourage such family communication. For both clinical care and research, there is little consideration of the risk of death prior to return of results and the patient's desire to share results in that instance. Among patients surveyed in a cancer biobank study, a majority (94%) favored a mechanism allowing their relatives to receive their genetic research results in the event of their death (Breitkopf et al. 2015). A Working Group of national experts addressing this issue recommended that patients be asked to specify their preference on how to share their data upon their death and to designate a representative for making decisions on sharing results (Wolf et al. 2015). We have previously shown that 92% of participants in one study designated an individual for return, while the reminder chose not to share this information in the case of their death (Amendola et al. 2015). We now explore whether the person designated for return is the same as the executor of the estate.

As part of the National Human Genome Research Institute (NHGRI) and National Cancer Institute (NCI) funded New Exome Technology in (NEXT) Medicine study, which offers randomization to exome sequencing for patients being evaluated for hereditary colorectal cancer/polyposis (CRCP), we developed a consent document that allows participants to designate a particular person to receive genomic results in the event of the participant's death prior to receipt. We have previously reported that 92% of these participants named a designee and that, when the designee was not a spouse, they were more often females (Amendola et al. 2015). In this paper, we report on whether the individual designated by patients participating in a genomic medicine research study to receive their exome sequencing results in the event of their death is the same as their named estate executor.

## Methods

Adult patients being evaluated for hereditary CRCP at the University of Washington, Seattle Cancer Care Alliance, or Group Health Cooperative genetic medicine clinics were recruited into the NEXT Medicine study (IRB # 41829). Participants agreed to be randomized to either usual clinical care (UC) or to UC plus exome sequencing and to the return of both diagnostic findings and select other findings including medically actionable additional (non‐colorectal cancer related) findings, pharmacogenomic results, and/or carrier status results. Although critically ill participants were not enrolled, during the course of the study, we recognized the risk of participant death prior to return of genetic results. Therefore, we adapted our informed consent process to ask participants at enrollment if, in the event of their death prior to receiving results, they would like their genomic results returned to a specified individual and, if so, whom. Those who said yes were asked to provide the designee's name, contact information (mailing address, phone number, and email address), and relationship with the participant. When we noted that women were disproportionately named, we hypothesized that these individuals were differing from any person named as estate executor and added this question to the enrollment consent process. Participant demographics, diagnosis, and family history information, including number of children, were collected directly from the participant and/or from the clinical genetics visit documentation in the electronic health record. A copy of the IRB approved consent form is available on the CSER website (https://cser-consortium.org/resources).

## Results

To date, 61 participants have been asked whether or not the individual they designated for return of their genomic results in the event of their death is the same as the individual who is the executor of their estate. The demographic characteristics of these participants are presented in Table 1.

**Table 1:** Demographic characteristics of participants

	N (%)
Ethnicity	
Hispanic/Latino	1 (2%)
Not Hispanic/Latino	60 (98%)
Self‐reported race	
White	52 (85%)
Asian	3 (5%)
American Indian/Alaska Native	6 (10%)
Sex	
Female	28 (46%)
Male	33 (54%)
Age	
18–50 years	22 (36%)
>50 years	38 (64%)
Education	
Graduate degree	13 (21%)
College degree	22 (36%)
Some college	16 (26%)
Completed vocational or trade school	3 (5%)
High school graduate	7 (12%)

### Participants with an estate plan

**Figure 1:**
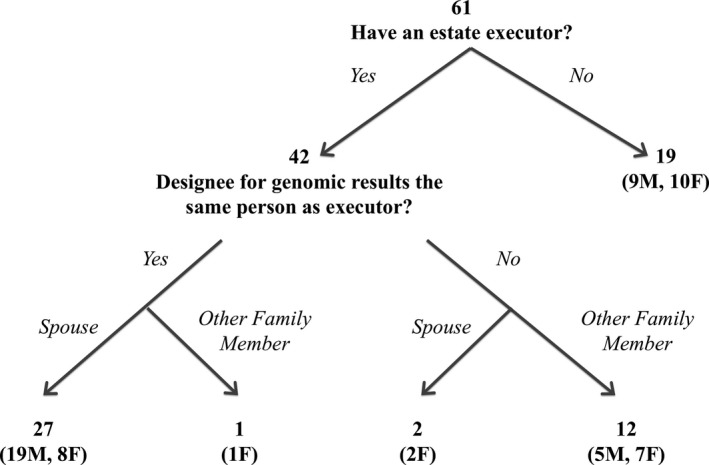
Participants with or without an estate executor and their designees for return of genomic results after their death.

A summary of participants with or without an estate executor and their designees for return of genomic results after their death is available in Figure 1. Of the 61 participants who chose a designee, 42 (69%) reported having a will or estate plan. Of these 42 participants, 14 (33%) had a different designee for their genomic data as their estate executor. Of these 14 participants who had a different genomic designee than their estate executor, two designated their spouse/partner and 12 a biological relative (five sisters, one brother, three daughters, two sons, one nephew). A majority of those who chose a different genomic designee than their estate executor chose a female designee (8/14; 57%). Of these 14 participants, nine were partnered and five were divorced or never married. The choices of these 14 participants are summarized in Table 2.

**Table 2:** Characteristics of participants with discordance in estate executor and designee for return of results

Gender of participant	Designee for genetic results	Estate executor	Marital status
F	Sister	Husband	Married
F	Sister	Other sister	Not married
F	Sister	Not disclosed	Married
F	Sister	Not disclosed	Not married
F	Partner	Not disclosed	Living with partner
F	Nephew	Husband	Married
F	Husband	Son in law	Married
F	Daughter	Son	Not married
F	Daughter	Not disclosed	Married
M	Son	Brother	Not married
M	Son	Fiancée	Engaged
M	Sister	Not disclosed	Married
M	Daughter	Not disclosed	Married
M	Brother	Not disclosed	Not married

The majority of those with the same person designated (27/28, 96%) selected their spouse/partner. The remaining individual designated the same person for both and chose a daughter. All of these 28 participants were partnered. Of the 37 partnered participants with an estate plan, 29 (78%) designated the partner to return genomic results to in the case of their death. Six of the remaining eight designated a female biological relative while the other two selected a male biological relative. Those with an estate plan who did not choose their executor for return of genomic results were less likely to be partnered; 9/14 partnered versus 28/28 of those with the same designee being partnered (*P* = 0.0024).

### Participants without an estate plan

Nineteen participants did not have an estate plan. This group of participants tended to be younger, with an average age of 47.8 years (range 24–72) versus 57.3 years (range 27–81) for those with an estate plan (*P* = 0.0025). Of the 14 partnered participants who did not have an estate plan, two selected someone other than their spouse for genetic data return; both selected adult children (one each daughter and son). The remaining five participants without an estate plan designated a biological family member (two brothers, one aunt, one mother, one sister). Family history for the 19 participants without an estate plan is as follows: nine had at least one child 25 years old or less, five had children older than 25 years of age (one of these had children in both categories), and six had no children. Thirteen of these participants were married, one was widowed living with partner, two were divorced, three never married.

## Discussion

The manner in which genetic information is shared after a person's death may have significant impact on families and it is thus important to understand a person's preference for sharing genetic results that become available after that person's death. In this circumstance, participants are unable to share the information, and family members may be unaware of the test and its potential relevance to their health. We have previously reported that participants who designated someone other than a spouse disproportionately selected female relatives to receive the information (Amendola et al. 2015). Given that estate executors are not disproportionately women, this led to the hypothesis that, particularly in the case of a nonspouse, the person named to receive genetic results may not be the same person designated to be the executor of the estate, that is, that the patient identifies different persons as suitable for these tasks. If so, the person deemed most suitable to have access to the genomic results by the participant may not have legal access to these data unless the participant is given the opportunity to name a designee.

Within the context of a genetic research study enrolling adults having clinical genetic testing for hereditary CRCP, 31% of participants did not have an estate plan, despite presenting for genetic cancer testing due to a compelling personal and/or family history of CRCP. Of those with an estate plan, 33% chose a person other than their estate executor to receive their genomic results in case of their death. Among 51 partnered participants, 20% selected a person other than their partner to receive the genomic data. Thus, neither executor nor partner status is an ideal indicator of who the participant feels is best able to act as recipient of genomic results. It is possible that the person chosen by these participants to share their genetic results is considered the best family medical information communicator, better able or equipped to navigate the emotional aspects that accompany sharing sensitive and possibly life‐changing information, but this individual may not necessarily be the same person considered the most appropriate to manage financial decisions after death. Obtaining consent to release information to the appropriate person requires study staff properly trained and prepared to talk with patients about sensitive information regarding their genetic results.

If a personal representative or estate executor is not explicitly designated, access to a person's health records after death is based on state law. While this varies by state, in many cases a spouse, adult child, or sibling may be considered the next of kin and can petition to be considered the personal representative. As previously noted, this approach may contradict the wishes of the participant and therefore highlights the importance of a mechanism to designate a person to receive genetic testing results in the event of their death.

Limitations of this project include the small sample size focused on a particular phenotype (CRCP) in the setting of a cancer genetic research study. Given the wide range of circumstances for which genomic sequencing is currently pursued, these results may not be generalizable to other populations. Furthermore, the majority of our study population was of European ancestry and receiving care in an academic medical setting; research evaluating these questions in different populations and health care settings is needed. We did not ask the participants why they chose the designee, nor did we address instances other than death, such as when a person becomes incapacitated and unable to make their preferences known.

The lack of an estate plan in 31% of those tested and the existence of discordance between the executor and the person named to receive genetic results, particularly in unpartnered participants, suggests the importance of a conversation with patients undergoing genetic tests about sharing these results with families, in the case of the patient's death prior to receiving results. No standard mechanism exists to allow patients to designate someone different than their estate executor or personal representative under HIPAA guidelines. Given the previously reported minority of participants who decline family sharing (Amendola et al. 2015) and that family members may not know that there is a genetic result to seek, such conversation and documentation best allow that patient/participant's wishes to be carried out.

## Disclosures

None.
